# Hypertriglyceridemic waist identifies HIV+ men and women at increased cardiometabolic risk

**DOI:** 10.1186/1758-2652-13-S4-P73

**Published:** 2010-11-08

**Authors:** G Guaraldi, S Zona, G Orlando, F Carli, C Stentarelli, J Despres, R Ross

**Affiliations:** 1 University of Modena and Reggio Emilia, Modena, Italy; 2 Université Laval, Ville de Québec, Canada; 3 Queen’s University, Kingston, Canada

## Background

Screening for increased waist circumference and hypertriglyceridemia (the hypertriglyceridemic-waist phenotype) is an inexpensive approach to identify patients at risk of coronary artery disease in apparently healthy individuals who may be at increased risk of type 2 diabetes and coronary heart disease because of an excess of intra-abdominal (visceral) fat. We examined the relationship between the hypertriglyceridemic-waist and selected cardiometabolic risk factors in HIV individuals.

## Methods

The HW phenotype was defined as a waist circumference of 90 cm or more and a triglyceride level of 2.0 mmol/L or more in men, and a waist circumference of 85 cm or more and a triglyceride level of 1.5 mmol/L or more in women. Using these threshold values a total of 2322 patients (841 women and 1481 men) with HIV aged 18-75 years were divided into 4 groups: Low TG/Low WC, High TG/Low WC, Low TG/High WC, High TG/High WC. Continuous variables were analyzed using ANOVA or Kruskal-Wallis test where appropriate; categorical variables were compared using X2-test. The relationship between the HW and cardiometabolic risk assessed with Framingham risk score (FRS) was analyzed using multivariable logistic regression analyses.

## Results

Compared with patients who had a waist circumference and triglyceride level below the threshold values, those with the HW phenotype had higher visceral adipose tissue (P<0.001), higher prevalence of hypertension and the metabolic syndrome (P<0.001), higher levels of total and LDL-cholesterol (P<0.001), lower levels of high-density lipoprotein cholesterol (P<0.001), and higher values of HOMA-insulin resistance (P<0.001) as shown in Table 1.

**Table 1:** 

	Low TG/Low WC	High TG/Low WC	Low TG/High WC	High TG/High WC	P value
n (%)	592 (25.50)	856 (36.86)	311 (13.39)	563 (24.25)	-

DEMOGRAPHICS					

Women, n (%)	284 (47.9)	245 (28.62)	145 (46.62)	167 (29.66)	< 0.001

Age mean (± S)	43.3 (6.7)	44.4 (6.6)	45.4 (7.7)	46.9 (7.8)	< 0.001

Physical activity, n (%)	232 (39.19)	282 (32.94)	103 (33.12)	152 (27.00)	< 0.001

Smoke (> 10 cigs/day), n (%)	187 (31.59)	285 (33.29)	73 (23.47)	165 (29.31)	0.010

Alcohol consumption, n (%)	270 (45.61)	363 (42.41)	154 (49.52)	279 (49.56)	0.032

ANTHROPOMETRICS					

BMI mean (± SD)	21.2 (2.2)	21.7 (2.3)	26.3 (3.9)	27.1 (3.8)	< 0.001

VAT cm^3^, median (IQR)	75 (49; 103)	100 (67; 138)	136 (101; 194)	172 (125; 236)	< 0.001

Waist Circumference cm, median (IQR)	79 (75; 83)	81 (77; 85)	94 (90; 98)	95 (91; 101)	< 0.001

Hip Circumference cm, median (IQR)	87 (83; 90)	86 (83; 89)	94 (91; 97)	94 (90.5; 98)	< 0.001

Thigh Circumference cm, median (IQR)	45 (42.5; 48)	45 (43; 48)	49 (46; 52)	49 (46; 52)	< 0.001

% of Leg Fat, median (IQR)	12.6 (7.2; 19.9)	7.7 (5.6; 12.7)	18.1 (12.5; 26.2)	14.0 (9.9; 21.1)	< 0.001

HIV history					

IDU n (%)	201 (33.95%)	286 (33.41%)	99 (31.83%)	151 (26.82%)	< 0.001

CD4+ Nadir median (IQR)	181 (78; 260)	154 (59; 260)	189 (90; 290)	171.5 (63; 260)	0.014

CD4+ Current median (IQR)	499.5 (370; 672)	523 (364; 700)	492 (360; 658)	543.5 (375; 737)	0.113

VL undetectable n (%)	363 (61.32)	492 (57.48)	190 (61.09)	329 (58.44)	0.432

Months of PI exposure median (IQR)	24 (0; 60)	35.5 (8; 69.5)	30 (0; 58)	39 (9;7269)	0.005

Months of NNRTI exposure median (IQR)	16 (0; 45)	18 (0; 48)	17 (0; 49)	16 (0; 46)	0.445

CARDIOVASCULAR					

Framingham risk median (IQR)	2 (1; 5)	6 (2; 10)	2 (1; 6)	6 (2; 12)	< 0.001

Hypertension, n (%)	131 (22.13)	302 (35.28)	119 (38.26)	259 (46.00)	< 0.001

Albuminuria, n (%)	38 (6.42)	84 (9.81)	24 (7.72)	59 (10.48)	< 0.001

LIPID METABOLISM					

Triglycerides median (IQR), mmol/L	1.03 (0.81; 1.27)	2.32 (1.87; 3.41)	1.10 (0.89; 1.30)	2.37 (1.85; 3.34)	< 0.001

Total cholesterol mean (± SD), mmol/L	4.43 (1.09)	5.05 (1.22)	4.55 (1.10)	5.17 (1.30)	< 0.001

HDL mean (± SD), mmol/L	1.36 (0.44)	1.05 (0.42)	1.33 (0.41)	1.06 (0.30)	< 0.001

LDL mean (± SD), mmol/L	2.70 (0.84)	3.02 (1.03)	2.83 (0.91)	3.09 (1.01)	< 0.001

ApoA1 mean (± SD), mg/dL	148.7 (32.6)	137.5 (26.8)	149.9 (29.8)	139.7 (27.0)	< 0.001

ApoB mean (± SD), mg/dL	85.6 (23.4)	108.8 (29.0)	90.3 (24.3)	110.3 (27.3)	< 0.001

HOMA-IR median (IQR)	2.25 (1.39; 3.38)	3.07 (2.04; 5.01)	3.26 (2.21; 5.13)	4.31 (2.74; 6.68)	< 0.001

**Figure 1:**
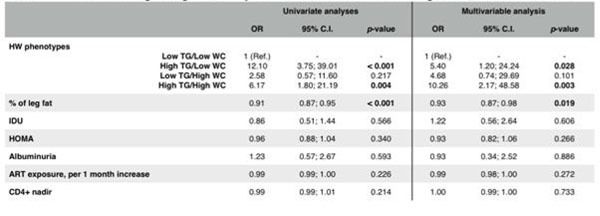
Univariate and multivariable logistic regression anlyses for associated factors with Framingham risk score more than 20%.

The FRS (median 10, range 5;16) was also highest in those with the HW phenotype (P<0.001). These observations were true independent of gender and remained significant after statistical control for illicit drug use, insulin resistance, antiretroviral therapy exposure, leg fat, and proteinuria as shown in image 1. Figure 1

## Conclusions

Among HIV patients from an Italian monocentric cohort, the HW phenotype was associated with a deteriorated cardiometabolic risk profile and an increased FRS. It is suggested that the simultaneous measurement and interpretation of waist circumference and fasting triglyceride could also be used among HIV patients as an inexpensive tool to identify patients with excess visceral fat and with related cardiometabolic abnormalities.

